# Diagnosis of secondary amyloidosis in alkaptonuria

**DOI:** 10.1186/s13000-014-0185-9

**Published:** 2014-09-26

**Authors:** Lia Millucci, Lorenzo Ghezzi, Giulia Bernardini, Daniela Braconi, Pietro Lupetti, Federico Perfetto, Maurizio Orlandini, Annalisa Santucci

**Affiliations:** Dipartimento di Biotecnologie, Chimica e Farmacia, Università degli Studi di Siena, via Aldo Moro 2, 53100 Siena, Italy; Dipartimento di Scienze della Vita, Università degli Studi di Siena, via A. Moro 2, 53100 Siena, Italy; Centro di Riferimento Regionale per lo Studio dell’Amiloidosi, Dipartimento di Medicina Sperimentale e Clinica, viale Pieraccini 18, Università degli Studi di Firenze, 50139 Firenze, Italy

**Keywords:** Serum amyloid A, Ochronosis, Secondary amyloidosis, Salivary gland, Periumbelical fat, Congo Red

## Abstract

**Background:**

Alkaptonuria (AKU) is an inborn error of catabolism due to a deficient activity of homogentisate 1,2-dioxygenase. Patients suffer from a severe arthropathy, cardiovascular and kidney disease but other organs are affected, too. We found secondary amyloidosis as a life-threatening complication in AKU, thus opening new perspectives for its treatment. We proved that methotrexate and anti-oxidants have an excellent efficacy to inhibit the production of amyloid in AKU model chondrocytes. Owing to the progressive and intractable condition, it seems important to detect amyloid deposits at an early phase in AKU and the choice of specimens for a correct diagnosis is crucial.

**Methods:**

Ten AKU subjects were examined for amyloidosis; abdominal fat pad aspirates, labial salivary gland, cartilage and synovia specimens were analysed by CR, Th-T, IF, TEM.

**Results:**

Amyloid was detected in only one abdominal fat pad specimen. However, all subjects demonstrated amyloid deposition in salivary glands and in other organ biopsies, indicating salivary gland as the ideal specimen for early amyloid detection in AKU.

**Conclusions:**

This is, at the best of our knowledge, the first report providing correct indications on the diagnosis of amyloidosis in AKU, thus offering the possibility of treatment of such co-morbidity to AKU patients.

**Virtual slides:**

The virtual slide(s) for this article can be found here: http://www.diagnosticpathology.diagnomx.eu/vs/13000_2014_185

## Background

Amyloidosis is the deposition of insoluble protein aggregates either systemically or in a specific organ. Secondary (AA) amyloidosis is caused by a chronic infection or chronic inflammatory disease where deposits are made up of serum amyloid A (SAA) produced during inflammation.

We found SAA amyloidosis as a life-threatening complication in alkaptonuria (AKU) [1], an ultra-rare (1:250.000-1.000.000 incidence) autosomal recessive genetic disease due to a deficient activity of homogentisate 1,2-dioxygenase (HGD) leading to the accumulation of homogentisic acid (HGA). HGA-oxidized derivative benzoquinone acetic acid (BQA) forms deposits in the connective tissue, causing a pigmentation known as “ochronosis”, leading to dramatic organ damage. A severe form of arthropathy is the most common clinical presentation of AKU, but all the organs can be affected, too. AKU can be treated symptomatically during the early stages, whereas for end stages total joint and heart valve replacements may be required. Currently, there is no therapy for AKU, although a clinical trial with nitisinone is in progress.

AA amyloidosis is one of the most severe complications of several chronic rheumatic disorders [2,3] and is a secondary complication of AKU due to a chronic inflammatory status derived from HGA/BQA-induced oxidative stress [4-12]. SAA-amyloid in AKU co-localizes with ochronotic pigment [1], AKU is a complicated inflammatory multisystemic disease, and any body district expressing *HGD* may be affected [13].

Our findings on AKU as a novel AA amyloidosis opened new perspectives for its treatment. In fact, the suppression of SAA below 10 mg/L halts the progression of the disease and is associated with prolonged survival, reversal of amyloid deposition and recovery of organ function [14]. Methotrexate [1] and anti-oxidants [9] proved to have an excellent efficacy to inhibit the production of amyloid in AKU model chondrocytes, suggesting their introduction in AKU therapy.

Owing to AKU progressive and intractable condition, it is crucial to detect amyloid deposits at an early phase. As ascertained in other secondary amyloidosis, if SAA deposition cannot be stopped or delayed, serious life-threatening problems can arise [14]. Therefore, SAA amyloidosis should be diagnosed as early as possible and the clinicians treating AKU patients need an easy routine reproducible fast procedure that can be repeated at intervals.

In this work, ten AKU subjects were examined for amyloidosis in abdominal fat pad aspirates and labial salivary gland (LSG) biopsies. Amyloid was detected in only one abdominal fat pad specimen. However, all subjects demonstrated amyloid deposition in LSGs and in other organ biopsies. These results further support the concept that LSG may be the optimal specimen for early detection of amyloid in AKU and that LSG offers improved sensitivity in respect to fat pad aspirates in these patients.

This is, at the best of our knowledge, the first report providing correct indications on the diagnosis of amyloidosis in AKU, thus offering the possibility of treatment of such co-morbidity to AKU patients.

## Methods

Procedures were approved by Siena University Hospital and national Ethics (Comitato Etico Policlinico Universitario di Siena, number GGP10058, date 21/07/2010) in accordance with 1975 Helsinki Declaration, revised in 2000 (52nd WMA General Assembly, Edinburgh, Scotland, October 2000). Informed consent was obtained from all patients.

All reagents were from Sigma-Aldrich (St. Louis, MO), if not differently specified.

### LSG

After reviewing AKU patients’ medical history and laboratory values (Table 1 and 2), a clinical examination was completed and each subject underwent two biopsies of clinically normal-appearing tissue in the oral cavity under local anesthesia (2% lidocaine with 1:100,000 epinephrine). Specimen 1 was taken from the left lateral surface of the tongue, containing mucosal and muscle tissue. Specimen 2 was taken from the labial mucosa on the left side of the lower lip, containing at least 2 to 3 minor salivary glands. Both specimens were formalin fixed and analyzed for histopathologic evaluation of amyloid.

**Table 1:** **Alkaptonuria patients enrolled for the study and their characteristics**

**Features**	**Patient 1**	**Patient 2**	**Patient 3**	**Patient 4**	**Patient 5**	**Patient 6**	**Patient 7**	**Patient 8**	**Patient 9**	**Patient 10**
Age (years)	71	60	63	42	60	58	42	38	65	55
Sex	F	F	F	F	M	M	M	F	F	F
*Serology*										
SAA mg/L	117.70	87.14	97.44	18.16	25.35	25.44	25.13	16.13	28.46	69.90
HGA plasma level μmol/L	31.10	72.90	20.56	15.80	17.02	22.43	18.95	15.21	31.76	13.42
HGA urine level mmol/L	23.70	16.90	22.64	21.70	22.30	18.64	24.10	12.68	22.76	16.60
*Congo Red birefringence score*										
Abdominal fat	n.d.	-	-	-	-	-	-	-	n.d.	+
Salivary gland	+++	++	++	++	++	+++	++	+++	++	++
Synovia			+++							
Cartilage			+++		+++					
Aortic valve			+++		+++					
Prostatic stone					++					
*ThT fluorescence (+/−)*										
Salivary gland	*positive*	*positive*	*positive*	*positive*	*positive*	*positive*	*positive*	*positive*	*positive*	*positive*
Synovia			*positive*		*positive*					
Cartilage			*positive*		*positive*					
Aortic valve			*positive*							
Prostatic stone					n.d.					
*SAA immunohistology (+/−)*										
Salivary gland	*positive*	*positive*	*positive*	*positive*	*positive*	*positive*	*positive*	*positive*	*positive*	*positive*
Synovia		*positive*		*positive*		*positive*	*positive*			
Cartilage		*positive*	*positive*		*positive*	*positive*	*positive*			
Prostatic stone					*positive*					
Aortic valve			*positive*							
*SAP immunohistology (+/−)*										
Salivary gland	*positive*	*positive*	*positive*	*positive*	*positive*	*positive*	*positive*	*positive*	*positive*	*positive*
Synovia			*positive*		*positive*					
Cartilage			*positive*		*positive*					
Aortic valve			*positive*							
Prostatic stone					*positive*					
*TEM Amyloid abundance score*										
	+++	*+++*	*++*	*+++*	*+++*	*+++*	*++*	*+++*	*++*	*+*

F: female, M: male, n.d.: not determined. The patients were classified on the basis of Congo red intensity of staining (grades: +++, very intensive; ++, intensive; +, weak; −, no birefringence). The presence of amyloid deposits in LSG observed at TEM was evaluated as: +++ = very abundant, ++ = medium; + = faint. Positivity for ThT fluorescence and SAA and SAP immunohistology is also reported.

**Table 2:** **Clinical features of AKU patients**

**Patient #**	**Date of selection (month/year)**	**Peripheral neuropathy**	**Orthostatic hypotension**	**Ventricular and atrial dilatation**	**chronic bacterial infection**	**Spondylarthropathy**	**Glossomegaly**	**Surgery**
**1**	08/2011	y	y	y	n	y	y	Knee (r), shoulder (r) and hip (l) arthroplasty
**2**	10/2009	y	y	n	y	y	n	Knee (r, l), shoulder (r) and hip (r) arthroplasty
**3**	03/2012	n	y	n	n	y	n	Meniscus (r) surgical repair
**4**	04/2012	y	y	n	n	n	n	None
**5**	11/2012	y	y	n	y	y	n	Knee (l), and hip (l) arthroplasty
**6**	01/2013	y	n	n	y	y	n	Knee (r) arthroplasty
**7**	05/2012	n	y	n	n	n	n	None
**8**	02/2012	y	y	y	n	y	n	Knee (l) arthroplasty
**9**	03/2013	y	n	y	y	y	y	Knee (r) and tendon ankle fracture (l)
**10**	11/2011	y	y	n	n	y	y	Knee (r), and hip (l) arthroplasty

No patient was receiving immunosuppressive or chemotherapy treatment. y = yes; n = no; r = right; l = left.

### Abdominal fat

Patients underwent a subcutaneous abdominal fat biopsy via fine-needle aspiration. Skin and subcutaneous tissue around the umbilicus of the patients were anesthetized with 1% lidocaine. The fat was aspirated with an 18-gauge needle connected to a 20 ml syringe. At least two visible fragments of fat were placed on a glass slide and crushed into a single layer by pressing a second slide against the first. After the smears were dried in air at 50°C for at least 30 minutes and fixed with 20% formalin neutral buffer solution (Wako Pure Chemical, Osaka, Japan) for 5 minutes, these specimens were processed for the Congo Red staining.

### Cartilage and synovia

Articular cartilage fragments, from AKU subjects who underwent surgery for joint replacement, were placed in 5% formaldehyde. Samples were alcohol dehydrated and paraffin embedded.

### Aortic valve

Valve was obtained from an AKU subject who underwent biologic aortic valve replacement.

### Prostatic calculus

The stone was from the prostate gland of an AKU subject who underwent surgical exploration for calculus removal.

### Hematoxylin and Eosin (H&E) staining

Two sections (5 μm thickness) were obtained from each biopsy. Sections were stained with traditional hematoxylin/eosin (H/E) staining.

### Congo Red (CR) staining

Romhányi’s CR staining method modified according to Bély and Apáthy (2000) was adopted. 5 μm sections of fresh specimen were fixed in cooled 96% ethanol 10 min, rinsed in water, incubated in 1% CR for 40 min, washed in water, incubated 10 s in 1 mL 1% sodium hydroxide in 100 mL of 50% ethanol, incubated 30 s in Mayer's hematoxylin, sequentially washed in 50%, 75%, 95% ethanol, mounted and observed under a polarized light microscope (Zeiss Axio Lab.A1, Arese, Milano).

### Thioflavin T (Th-T) staining

AKU LSG samples incubated in 1% Th-T [15,16] were mounted and observed under a fluorescence microscope (excitation 450 nm, emission 482 nm).

### Fluorescence microscopy

AKU samples in paraffin were cut in 3–5 μm slices and double immunofluorescence stained with anti-SAA and anti-serum amyloid P (SAP) antibodies (Santa Cruz Biotechnology, CA). Nuclei were counter-stained with blue DAPI DNA stain (Abcam, Cambrige, UK).

### Transmission electron microscopy (TEM)

AKU specimens were fixed in 2.5% glutaraldehyde in 0.1 M cacodylate buffer (CB) pH 7.2 for 3 h at 4°C, post-fixed in 1% osmium tetroxide in CB for 2 hours at 4°C, ethanol dehydrated and embedded in a mixture of Epon–Araldite resins. Thin sections, obtained with a Reichert ultramicrotome, were stained with uranyl acetate and lead citrate and observed with a TEM FeiTecnai G2 spirit at 80 Kv.

## Results

**Figure 1:**
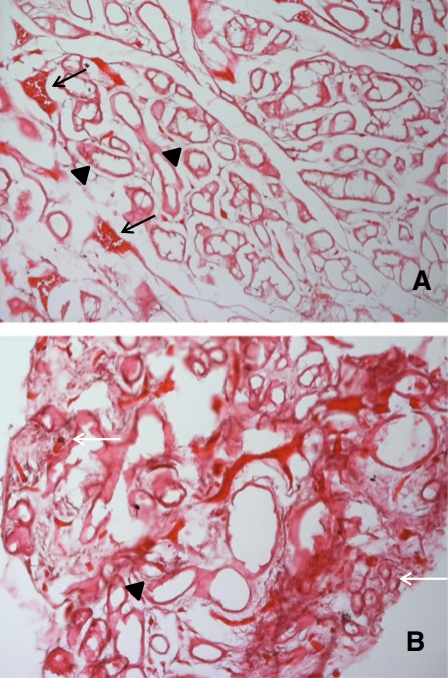
**H/E staining of AKU salivary gland.** Some striated ducts (arrowheads), secretory acini (white arrows) and red blood cells (black arrows) are indicated. Only representative images are shown. Magnification: **A**: 20×; **B**: 40 × .

Structural features of AKU salivary glands were first evaluated by means of H&E staining (representative images are presented in Figure 1A-B). Parenchyma was found to be compact only in some limited areas; by contrast, a number of vacuoles and a disrupted acinar structure were present in the vast majority of the specimen. All of the AKU salivary glands that were examined did not contain any trace of lymphocytic infiltration.

In order to diagnose amyloidosis in AKU patients, diagnostic laboratories adopted periumbelical fat aspirate CR staining [17] that unfortunately proved to be unsuitable for the detection of amyloid deposits in AKU patients. Indeed, we studied abdominal fat pad aspirates and labial salivary gland biopsies from 10 AKU patients in whom subsequent other tissue biopsies for confirmation of AA amyloidosis were also available. All cases were suspected to have SAA deposits, but nine of them had negative results on abdominal fat pad aspirates evaluated by polarizing microscopy of CR stained sections (not shown). CR staining of fat aspirates from three different hospital laboratories were compared in all samples for studying inter observer reproducibility and gave negative diagnosis of amyloidosis in nine cases out of ten.

**Figure 2:**
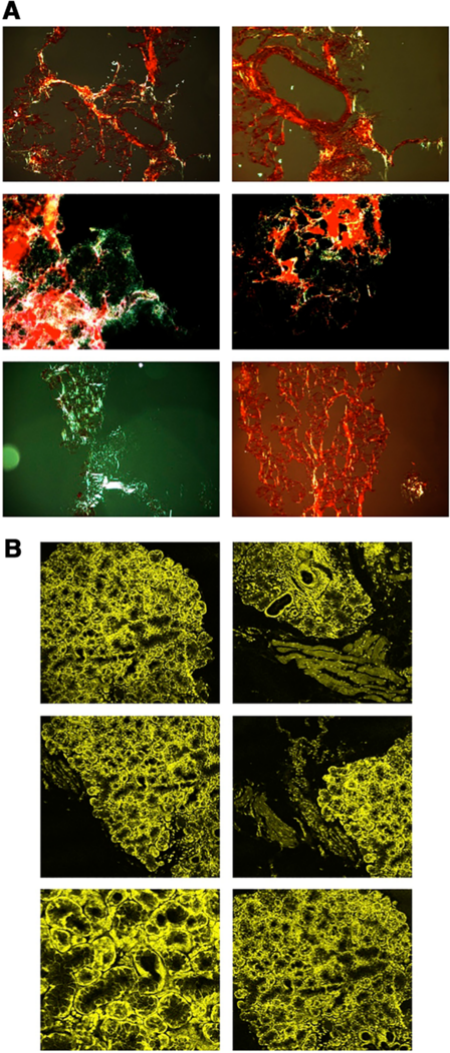
**Evidence of amyloid deposits in AKU salivary glands** ***.*** **A)** *Congo Red staining of AKU labial salivary glands amyloid deposits.* CR stain viewed under polarized light detected the presence of diffuse amyloid deposits within AKU salivary gland tissue. Magnification 20×. Representative images from a triplicate set are shown. **B)** *Th-T fluorescence of AKU labial salivary glands amyloid deposits shown by confocal microscopy.* Analogous results were obtained from specimens of other patients. Bar: 22 μm. Representative images from a triplicate set are shown.

Once CR staining was adopted on AKU LSGs, the results were unequivocally positive for all ten patients (Figure 2A).

**Figure 3:**
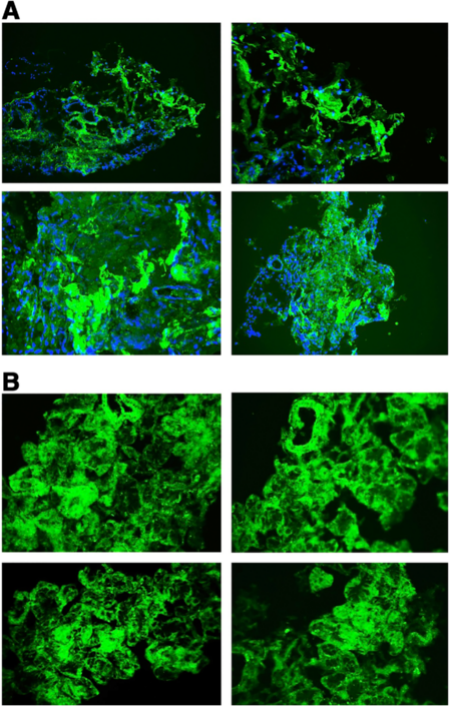
**SAA and SAP in AKU salivary glands** ***.*** **A)** *Immunofluorescence staining of SAA deposition in AKU labial salivary glands.* Blue indicates DAPI stained nuclei. Magnification 20×. Representative images from a triplicate set are shown. **B)** *Immunofluorescence staining of SAP deposition in AKU labial salivary glands.* Magnification 20×. Representative images from a triplicate set are shown.

To confirm the presence of amyloid aggregates in LSG from AKU patients, we performed the Th-T assay. Th-T fluorescence was evident in all examined samples (10/10) (Figure 2B). Moreover, by means of immunofluorescence techniques, we assessed the presence of SAA deposition in LSG (Figure 3).

**Figure 4:**
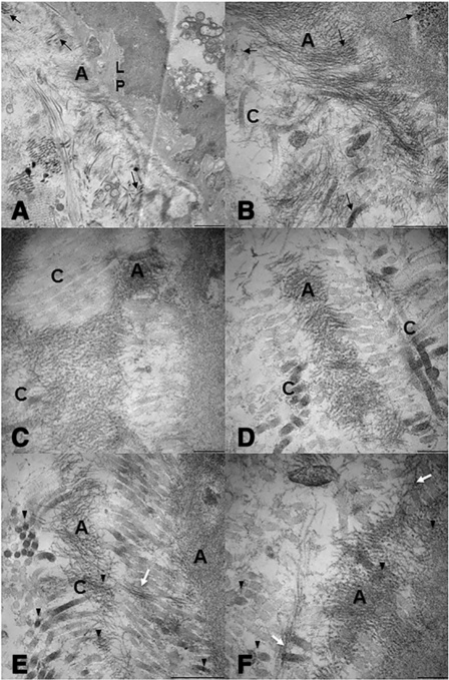
**Electron micrographs of AKU salivary glands** ***.*** **A)** Electron micrograph showing fine amyloid fibrils **(A)** approximately 10 nm in diameter located in close relation to the lamina (LP) of the secretory end-pieces as well as in the interstitial connective tissue stroma of AKU labial salivary gland. Pigment granules are visible amongst collagen fibrils in transverse and longitudinal section (black arrows). **B-C)** Electron micrographs of another AKU labial salivary gland showing a region of the interstitial glandular stroma that contains fine amyloid fibrils **(A)**, approximately 10 nm in diameter, interspersed with bundles of collagen fibrils **(C)**. Pigment deposits are present on broken collagen fibers (black arrows) and scattered between amyloid fibrils. To be note that amyloid fibrils appear superimposed to collagen fibers in different area of the tissue. **D-F)** Electron micrograph of a third AKU labial salivary gland showing the glandular stroma containing finely fibrillar amyloid material **(A)**. In photo **E)** collagen fibers in transverse section are shown (arrowheads), many of which presenting electron dense ochronotic deposits located amongst fibers. In photos **E-F)**, dark ochronotic pigment granules are indicated (arrowheads) scattered amongst the collagen fiber as well as amongst amyloid fibrils. Some deposits can be observed located within amyloid bundles of fibrils and bridging between collagen fibers (white arrows). A: amyloid; C: collagen; LP lamina propria. Photo **A** is from patient #2, photos **B-C** are from patient #5 and photos **D-F** are from patient #7. Bars, **A**: 5 μm, **B, C** and **E**: 2.5 μm, **D** and **F**: 1 μm.

Ultrastructural analysis by TEM of AKU LSGs (the current ‘gold standard’ for detecting amyloid deposits also in ambiguous cases), confirmed the massive presence of amyloid fibrils in all specimens. Amyloid deposits in LSG were abundant in six patients, moderate in three, and mild in one (Table 1). Figure 4 shows fine fibrils, approximately 10 nm in diameter, located in the secretory stroma of AKU salivary glands of patient #1, adjacent to the basement membrane. The fibrils were also present in the interstitial connective tissue, interspersed with collagen fibrils (Figure 4A) and around interlobular ducts of salivary glands of all patients. In all observed samples, the glandular stroma contained numerous broken collagen fibrils strictly interconnected with amyloid fibrils arranged in a scattered manner. Moreover, thanks to TEM analysis, it was possible to see evident pigment traces finely sprinkled over the whole tissue (Figure 4B-F). In particular, pigment deposits could be seen as individual “drop-like” deposits between the collagen fibers and also scattered over amyloid bundles of fibrils.

Our study showed, for the first time, that, analogously to other AKU tissues [1,9], also in LSG amyloid and pigment were strictly interconnected and that collagen fibrils seemed to feel the effect of destructive action of both pigment and amyloid, appearing constantly broken and damaged when sprinkled and invaded by these two elements. The presence of ochronotic pigmentation in less commonly examined areas of human body, such as glandular tissues, may provide insights into the mechanisms of pigment formation and deposition. Here, we presented evidence to support the probable association of HGA oxidised products with collagen and the existence of extracellular mechanisms, like as amyloid deposition, mediating ochronotic pigmentation that overlaps in tissues.

**Figure 5:**
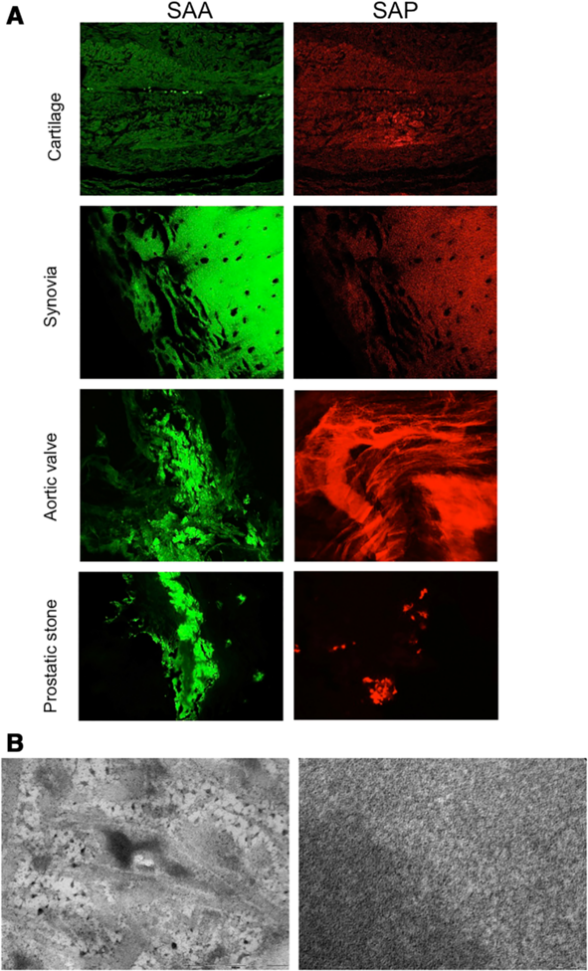
**Confirmation of amyloidosis in other AKU tissues** ***.*** **A)** Positive staining for SAA-and SAP-amyloid in cartilage, synovia, aortic valve and prostatic stone. **B)** TEM observation of amyloid deposits in cartilage (left image: bar 500 nm) and aortic valve (right image: bar 50 nm).

Confirmatory evaluation of SAA- and SAP-amyloid co-presence (Figure 5A) and TEM observation of amyloid fibrils (Figure 5B) in other AKU specimens was possible.

## Discussion

The tissue source impacts the likelihood of discovering amyloid deposits. Bone marrow biopsy sensitivity has been estimated at 63% [18]. Kidney, liver, or cardiac biopsies have sensitivity as high as 87–98% but are more invasive [18]. Rectal biopsy sensitivity ranges from 69% to 97% depending upon the quantity of tissue sampled. Tissue source also impacts amyloid typing. The chemical type of amyloid that deposits in AKU is amyloid A protein and since this type of amyloid involves diffusely the mucosa and the submucosa [19], it may be better to prefer location as the oral cavity or gastrointestinal tract to detect them. In particular, LSG biopsy is less invasive and cheaper than examination of rectal mucosa. In the last years, various studies have demonstrated the utility of LSG biopsy for the detection of amyloid with correlation to secondary systemic amyloidosis [20,21].

Amyloid deposition is a dynamic process that can progress and stabilize. Quantification of SAA concentration in tissue on regular occasions will similarly reflect the accumulation, stabilization or even regression of deposited amyloid. Abdominal subcutaneous fat tissue seems to be very suitable for this purpose [17,22], because it is easy to obtain by aspiration, but, at least in some cases, it has limited sensitivity and turned out inadequate [23-25]. In fact, subcutaneous abdominal fat CR staining is positive in approximately 80% of patients with AL amyloidosis and less than 65% of patients with AA amyloidosis [24]. Thus, the negative result on staining of the abdominal-fat aspirate in AKU cases does not necessarily indicate negativity of amyloidosis at the diagnosis. Moreover, Tribe [26] did not recommend the use of subcutaneous fat aspiration because he reported that “fat is rarely involved”. Libbey et al. [17] reported a 20% false negative result rate. It thus appears that abdominal fat aspiration is a method not enough sensitive to detect AA in rheumatic diseases.

In all ten AKU samples, amyloid deposits were identified so underlining the high sensitivity of LSG biopsy in the diagnosis of amyloidosis, even in the absence of other symptoms.

## Conclusions

LSG biopsy appears as a simple and safe method to detect generalized amyloidosis in patients with a chronic inflammatory disease such as AKU. The results of our pilot study demonstrate that the prevalence of occult amyloid deposition in patients with AKU may be very high. Since a prompt detection of amyloid may have significant clinical and economic implications in AKU, it is fundamental to establish accurately the association of synchronous amyloidosis. In our limited size sample, due to ultra-rarity of the disease, the majority of AKU patients had no detectable amyloid deposition on fat pad aspirate. However, we were able to detect amyloid deposition in minor LSG in all AKU patients. This suggests that subclinical amyloid deposition may be more easily detected on oral biopsies, and the oral cavity may be the preferred biopsy site for detecting amyloid deposition in AKU patients with no symptoms of systemic amyloidosis.

Our study suggests that minor LSG, especially when the biopsy of affected are lacking, may be the gold standard for the diagnosis of SAA amyloidosis in AKU.

## Abbreviations

AKU: Alkaptonuria
BQA: Benzoquinone acetate
CR: Congo red
HGA: Homogentisic acid
HGD: Homogentisate 1,2-dioxygenase
LSG: Labial salivary gland
SAA: Serum amyloid A
SAP: Serum amyloid P
Th-T: Thioflavin T
